# A longitudinal assessment of periodontal disease in Yorkshire terriers

**DOI:** 10.1186/s12917-019-1923-8

**Published:** 2019-06-21

**Authors:** Corrin Wallis, Ilaria Pesci, Alison Colyer, Lisa Milella, Peter Southerden, Lucy J. Holcombe, Neil Desforges

**Affiliations:** 1 The WALTHAM Centre for Pet Nutrition, Melton Mowbray, Leicestershire LE14 4RT UK; 2 The Veterinary Dental Surgery, Byfleet, Surrey KT14 7AA UK; 3 Eastcott Veterinary Clinic and Hospital, Swindon, Wiltshire SN3 3FR UK

**Keywords:** Dog, Canine, Gingivitis, Periodontitis, Periodontal disease, Yorkshire terrier

## Abstract

**Background:**

Periodontal disease is the most common oral disease of dogs and has been associated with systemic disease. The purpose of the present study was to determine the extent of periodontal disease in a population of Yorkshire terrier dogs with and without a tooth brushing regimen. Each dog was assessed under general anaesthesia two to five times between 37 and 78 weeks of age. The extent of gingivitis and periodontitis was ascertained for every tooth in the mouth. Gingivitis was measured using time to bleeding on probing, and periodontitis was based on extent of clinical attachment loss (probing depth, gingival recession and furcation exposure)**.**

**Results:**

Of the 49 dogs assessed at 37 weeks of age, 98% had at least one tooth or aspect with early periodontitis (PD2, < 25% attachment loss). The average percentage of teeth with periodontitis in the mouth was 29.6% with 95% confidence interval (23.6, 36.4). The odds of early periodontitis was 2.74 (2.23, 3.37) times higher at 78 weeks of age compared to 37 weeks of age. The canine teeth had a significantly higher probability of periodontitis compared to all other tooth types at both 37 and 78 weeks of age (*p* < 0.001). In addition, at the same time points, the incisors had a significantly higher probability of periodontitis compared to the molars and premolars (*p* < 0.001).

**Conclusions:**

Breeds of dog that are susceptible to developing periodontitis, such as Yorkshire terriers, require effective treatments for the prevention of periodontal disease from a young age. Although tooth brushing is one of the most effective methods when it comes to preventative homecare, this is not always realistic, as was found in this study. Therefore alternative ways to retard or prevent plaque accumulation that are practical for both dogs and their owners are required.

**Electronic supplementary material:**

The online version of this article (10.1186/s12917-019-1923-8) contains supplementary material, which is available to authorized users.

## Background

Periodontal disease is the most common oral disease of dogs with prevalence estimates ranging between 44 and 100% [1–5]. The incidence and severity of the disease has been shown to increase with age [3–5]. It is a progressive disease and the initial stage gingivitis is reversible and this may progress to periodontitis, which is irreversible but often controllable. Gingivitis is inflammation of the gingiva whereas periodontitis is characterised by apical migration of gingival attachment, destruction of the periodontal ligament, alveolar bone loss and potentially tooth loss [6]. Primarily periodontal disease is initiated by plaque accumulation on the surfaces of the teeth, but behavioural, environmental, systemic and genetic factors may also contribute to a dog’s susceptibility and clinical expression [7]. Periodontal disease has also been linked with renal, hepatic and cardiac disorders [8, 9].

Differences in the prevalence and severity of periodontal disease have been reported with smaller breeds of dog found to be particularly susceptible compared to larger breeds. Smaller dogs have been reported to have more calculus, gingival inflammation, furcation exposure and attachment loss, and an earlier onset of disease, than larger dogs [2, 4, 5, 10, 11]. Some of the most affected breeds include Yorkshire terriers, toy and miniature poodles, dachshunds cocker spaniels and Jack Russell terriers [4, 11]. Some of the least affected breeds include border collies, German shepherd dogs, Labrador retrievers and Staffordshire bull terriers [11].

Although there have been a few studies on the prevalence of periodontal disease in purebred dogs, further studies are still required to understand which breeds are most at risk. Most studies have focussed on the prevalence of the disease, and further research is necessary to understand the progression of the disease over time in different breeds of dog. A recent study of miniature schnauzers showed that in the absence of an oral care regimen, periodontal disease developed rapidly, with 28.3% of the teeth assessed progressing to periodontitis over the course of 60 weeks [12]. Understanding the extent and rate of progression of periodontal disease in different breeds of dog will enable veterinarians to understand the risk for a particular breed and provide individualised treatment and prevention strategies. The purpose of the present study was to determine the extent of gingivitis and periodontitis, and the distribution of teeth affected, in a population of Yorkshire terrier dogs tracked from 37 to 78 weeks of age with and without a tooth brushing regimen.

## Results

Of the 50 Yorkshire terriers that were recruited onto the study, 22 dogs (44%) came off trial at their first dental assessment at 37 weeks of age due to the presence of periodontitis in 12 or more teeth (Additional file 1: Table S1). A further 21 dogs (42%) came off trial at their second dental assessment at 45 weeks of age, six (12%) at their third dental assessment at 53 weeks of age, and one (2%) at their fourth dental assessment at 61 weeks of age. This meant that for some dogs only the prevalence of periodontitis at 37 weeks of age could be determined whereas for others it was also possible to determine the time to periodontitis. The high prevalence of periodontitis at baseline (37 weeks of age) prompted the decision to re-assess a subset of the dogs approximately a year later. Of the 50 dogs recruited to the study, 36 (72%) had their periodontal health status re-assessed at 78 weeks of age.

One dog missed the 37 week dental assessment, and another had the 53 week dental 3 weeks late due to not being able to insert an intravenous catheter on the scheduled dental date. Four dogs, from the first two litters recruited to the study, were not removed from trial when they had met the study criteria of developing periodontitis in 12 or more teeth because initially it was not clear if the probing depths measured were due to partial eruption of their permanent dentition. However, subsequent dental radiographs and consultation with a Diplomate of the European Veterinary Dental College (EVDC) (Lisa Milella) confirmed that there was indeed bone loss and that the probing depths were the result of periodontitis and therefore their respective data were used in the analyses. At this point dogs received a full-mouth professional dental cleaning (scale and polish) and were removed from the study.

Analysis of the probability of successful tooth brushing using generalised linear mixed effects models (GLMM) found a significant effect of week with an increase of success from 37 weeks of age (*p* < 0.0001). However, the probability of success for an average week during the study period was low at 3.99% with 95% confidence interval (0.05, 78.52). In addition, an interim analysis of data from 21 dogs who remained on the study beyond 37 weeks of age, to enable an estimation of the number of dogs required to detect a minimum effect size of a 5 week difference in time to periodontitis for this study, showed that the time to periodontitis for a tooth or aspect did not significantly differ between tooth brushing groups. There was approximately half a weeks’ difference in the time to periodontitis for both the tooth and aspect models, with a confidence interval width of approximately +/− 5.5 weeks. For both these reasons tooth brushing was dropped from the study design and from subsequent statistical analysis models.

### Periodontal health status at 37 weeks of age

**Fig. 1:**
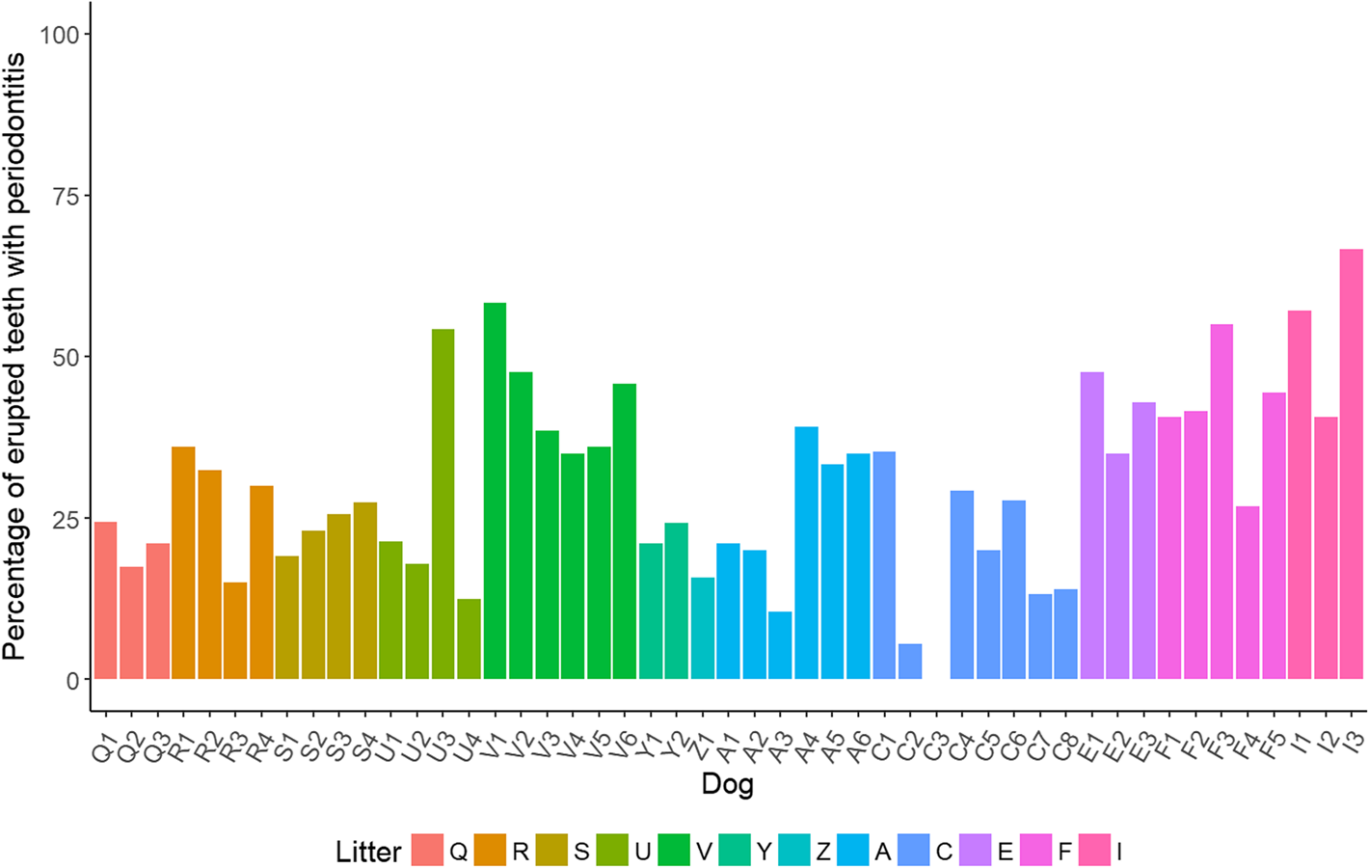
Bar chart of the percentage of erupted teeth with periodontitis at 37 weeks of age, by dog, coloured by litter

**Fig. 2:**
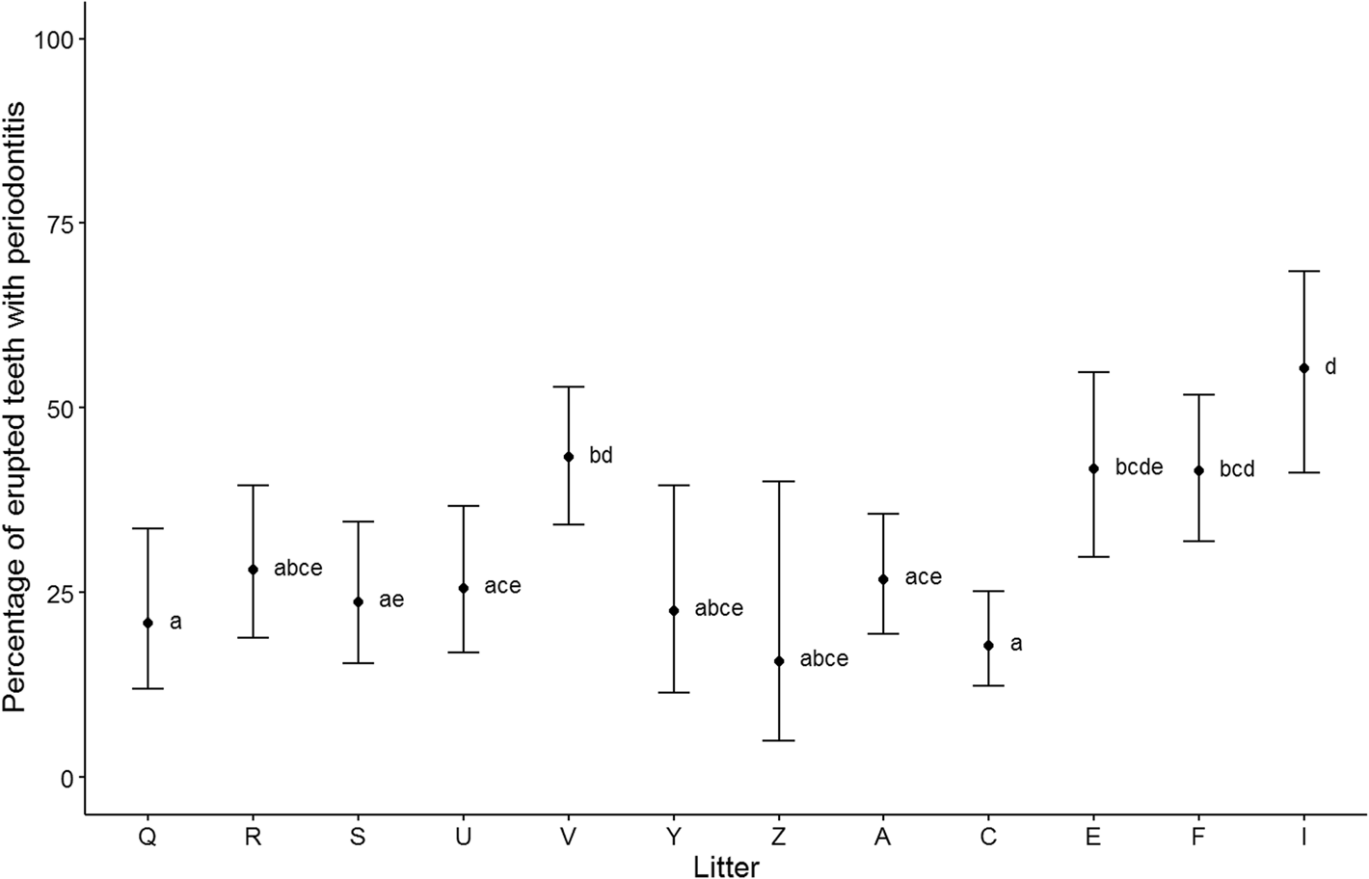
Average percentage of erupted teeth with periodontitis at 37 weeks of age by litter, with 95% confidence intervals and Tukey homogeneous groups at the 5% level

At 37 weeks of age, 48 of the 49 dogs assessed (98%) had periodontitis in one or more teeth. Of the 1874 erupted teeth at 37 weeks of age, 571 had periodontitis (30.5%). The number of teeth per dog with periodontitis ranged from zero to 24 (0–67%; Fig. 1). All teeth with periodontitis were classified as PD2 (early periodontitis, < 25% attachment loss) with the exception of three canine teeth which were classified as PD3 (moderate periodontitis, 25–50% attachment loss). Statistical analysis using a GLMM estimated that the average percentage of periodontitis teeth in the mouth at 37 weeks of age was 29.6% with 95% confidence interval (23.6, 36.4). The average percentage of periodontitis teeth in a mouth was significantly different across litters and varied from 15.8% (5, 40.1) to 55.3% (41.3, 68.6) (Fig. 2). For example, one litter (I) had a significantly higher percentage of periodontitis teeth in the mouth compared to eight of the other litters with odds ratios ranging from 3.2 (1.3, 7.6) to 6.6 (1.3, 32.3), *p* < 0.05. Litter C had a significantly lower percentage of periodontitis teeth in the mouth than four other litters with odds ratios ranging from 3.2 (1.7, 6.4) to 5.7 (2.5, 12.8), *p* < 0.001.

### Time to periodontitis

A total of 1914 teeth were present at the start of the study or erupted before 61 weeks of age. Between 37 and 61 weeks of age a total of 875 teeth (45.7%) were classified with periodontitis. Consequently, a number of dogs were removed from the trial early to conform with the requirements of the WALTHAM Animal Welfare and Ethical Review Body (AWERB) (Table 1). This resulted in a total of 1039 censored teeth that were unable to be followed to determine if they would progress to periodontitis. All the teeth were classified as PD2 with the exception of two incisors at 53 weeks from one dog.

**Table 1:** Number of teeth that developed periodontitis at each time-point, number of dogs that were removed from study as a result of developing periodontitis in 12 or more teeth and consequently number of teeth that did not have the opportunity to progress to periodontitis (censored)

Age (weeks)	Number of periodontitis teeth	Number of dogs removed from study	Number of censored teeth
37	571	22	476
45	234	21	449
53	63	6	97
61	7	1	17
78	702	*	*

*Not applicable as this was the last measurement and all dogs were taken off trial at this point and given a full mouth scale and polish

**Fig. 3:**
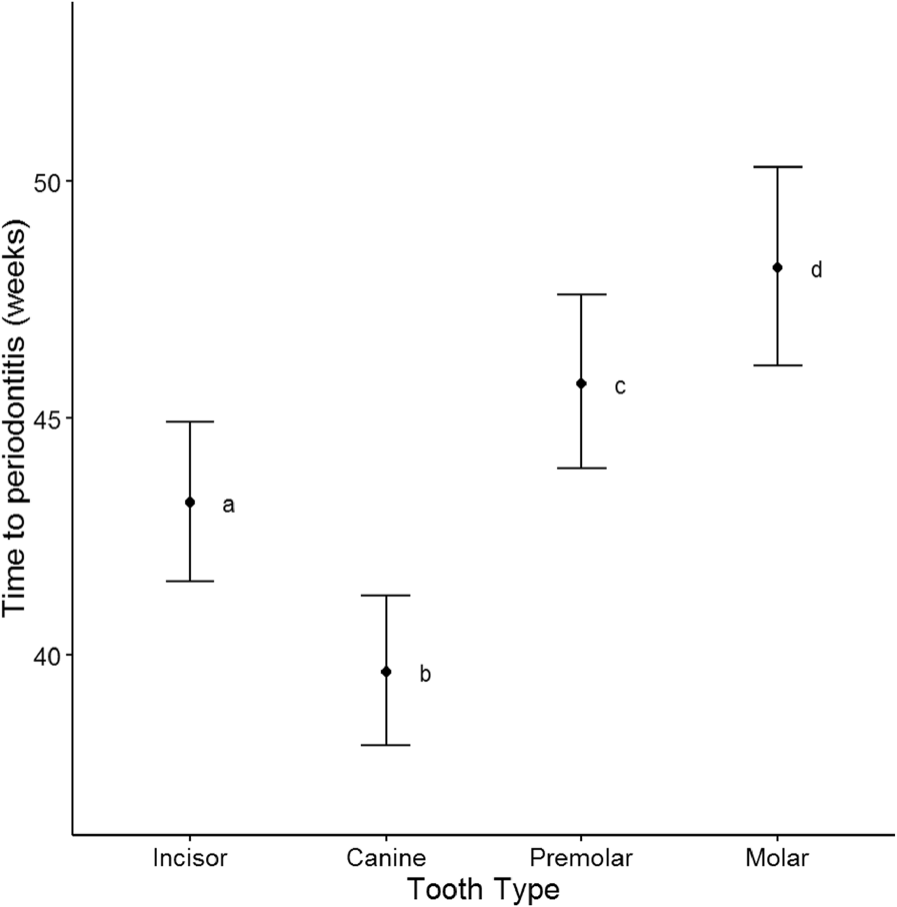
Average time to periodontitis for a tooth in weeks by tooth type, with 95% confidence intervals and Tukey homogeneous groups

Estimates of the time to periodontitis using statistical modelling of the tooth data showed that the canine teeth had a significantly quicker time to periodontitis than all other tooth types (*p* < 0.001; Fig. 3). The time to periodontitis for the canines was 39.6 weeks (37.9, 41.4) compared to 43.2 weeks (41.3, 45.2) for the incisors, 45.7 weeks (43.7, 47.9) for the premolars, and 48.2 (45.8, 50.6) for the molars. Incisors had a significantly quicker time to periodontitis when compared to the molars and premolars by 2.5 to 5.0 weeks (*p* < 0.001). Premolars had a significantly quicker time to periodontitis when compared to the molars by 2.4 weeks (*p* < 0.001). The maxillary teeth had a significantly (*p* < 0.001) quicker time to periodontitis when compared to the mandibular teeth, from 43.5 weeks (41.7, 45.5) to 44.6 weeks (42.7, 46.7). It was estimated that there was a significant decrease (*p* < 0.001) in time to periodontitis of 1.1 weeks (0.4, 1.8) for every unit increase in average gingivitis score.

**Fig. 4:**
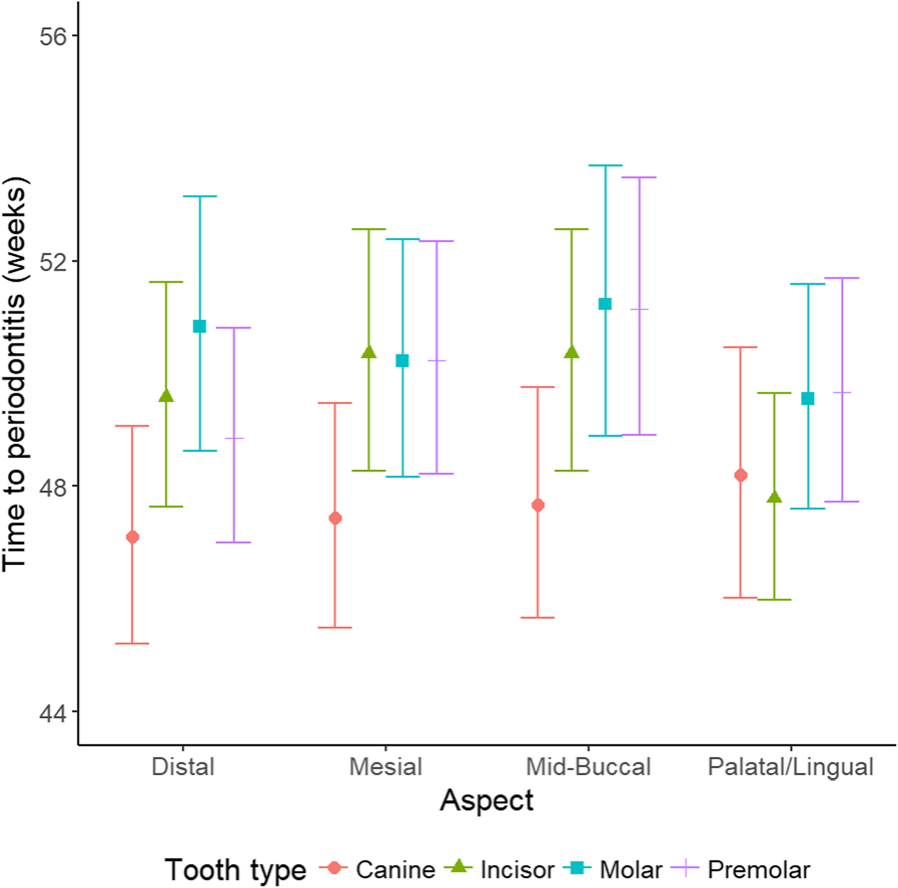
Average time to periodontitis on an aspect in weeks by tooth type with 95% confidence intervals

Estimates of the time to periodontitis for an aspect showed that the canine teeth had a significantly quicker time to periodontitis than other tooth types on all but the palatal/lingual aspect by 1.8 to 3.7 weeks (*p* < 0.001: Fig. 4). The mean times to periodontitis in weeks for the canine tooth on the distal, mesial and mid-buccal aspect were 47.1 (45.2, 49.1), 47.4 (45.4, 49.5) and 47.7 (45.7, 49.5), respectively, compared to between 48.9 (47.0, 50.8) and 51.1 (48.9, 53.5) for the other tooth types. The incisors progressed significantly faster to periodontitis by 1.8 to 1.9 weeks when compared to the molars and premolars on the palatal/lingual aspect (*p* < 0.001). The time in weeks to periodontitis was 47.8 (46.0, 49.7) on the palatal/lingual aspect of the incisors compared to between 48.2 (46.0, 50.5) and 49.7 (47.7, 51.7) for the other tooth types. The distal aspect of the premolars had a significantly quicker time to periodontitis when compared to the molars, from 48.9 (47.0, 50.8) to 50.8 weeks (48.6, 53.1), a difference of 1.9 weeks. The time to periodontitis was significantly (*p* < 0.001) quicker on the maxilla, 49.1 weeks (47.3, 51.1), compared to the mandible, 49.6 weeks (47.7, 51.6).

### Periodontal health status at 37 weeks compared to 78 weeks of age

**Fig. 5:**
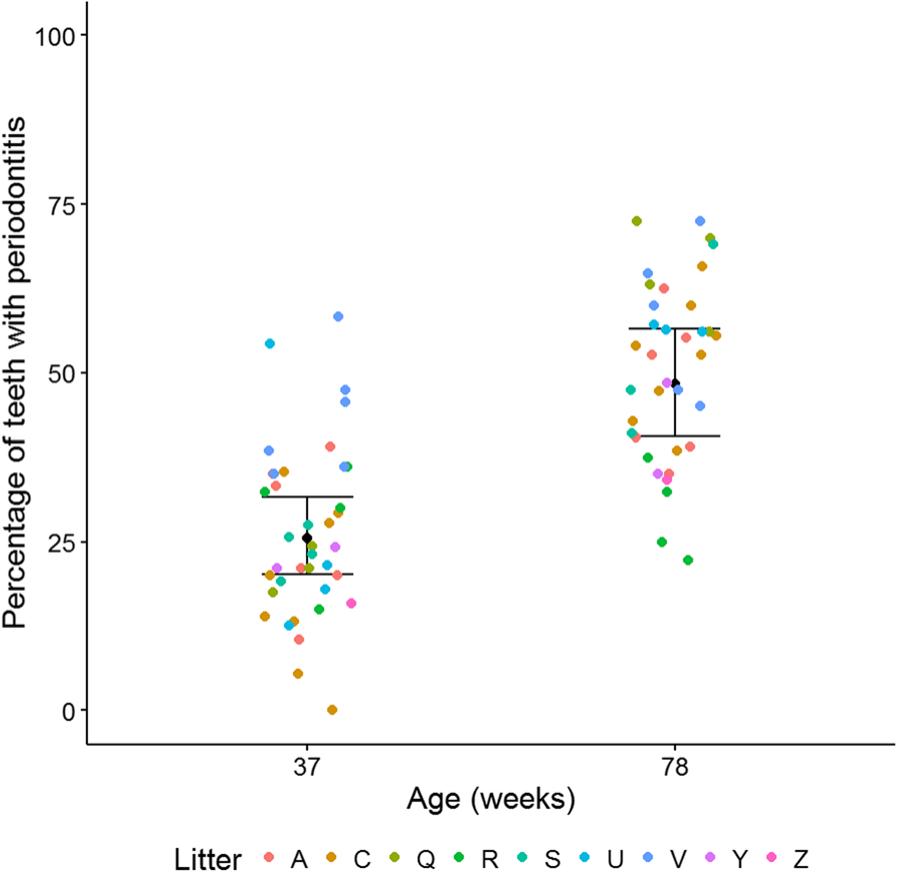
Percentage of teeth with periodontitis by age. Data are coloured by litter and averages are shown with 95% confidence intervals

For 36 dogs, an estimation of the probability of periodontitis in the mouth at 37 weeks of age compared to 78 weeks using statistical modelling showed that the odds of periodontitis in a mouth at 78 weeks was 2.74 times that at 37 weeks of age with 95% confidence interval (2.23, 3.37, *p* < 0.001). The average percentage of periodontitis teeth at 37 weeks was 25.5% (20.6, 31.1) compared to 48.3% (41.4, 55.4) at 78 weeks (Fig. 5). All teeth with periodontitis at 78 weeks of age were classified as PD2 with the exception of one incisor from one dog which was deemed to be PD3. The odds of periodontitis in the mouth increased by 1.6 times (0.94, 2.72) for each unit increase in gingivitis but this was not significant after adjustment for multiplicity (*p* = 0.088).

**Fig. 6:**
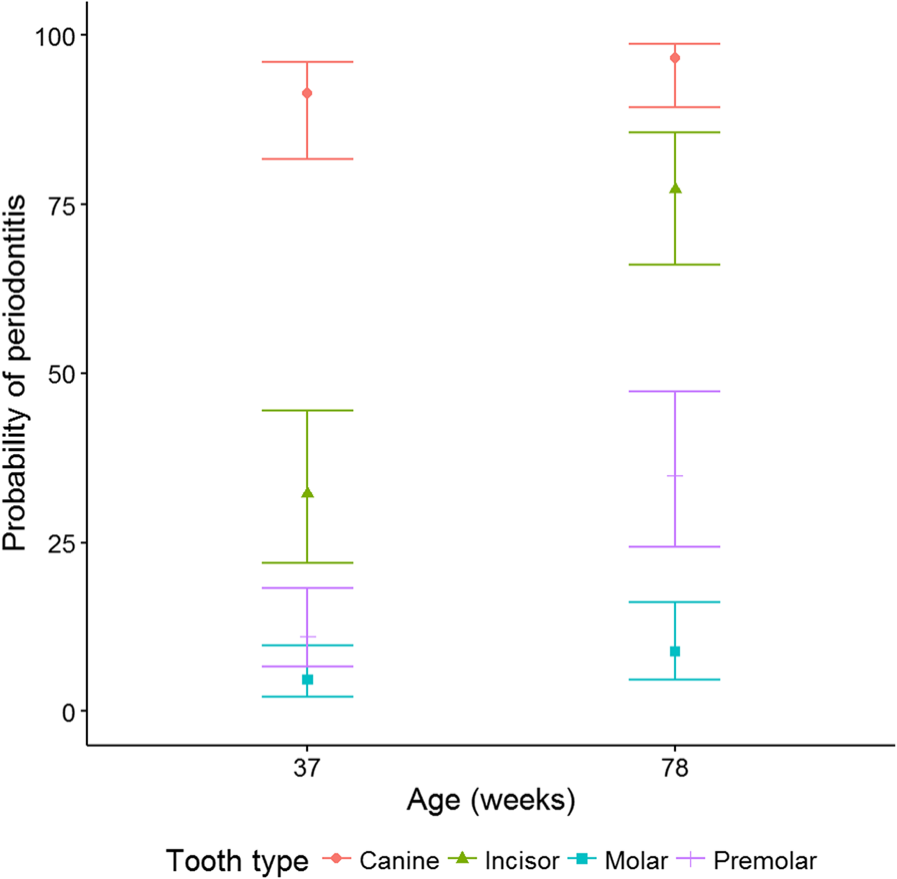
Probability of periodontitis on a tooth at 37 and 78 weeks of age by tooth type, with 95% confidence intervals

Estimation of the probability of periodontitis for a tooth at 37 weeks compared to 78 weeks was explored using statistical modelling. This showed that the canine teeth had a significantly higher probability of periodontitis compared to all other tooth types at both 37 and 78 weeks of age, 91.4% (83.0, 94.8) and 96.7% (91.2, 96.9), respectively, (*p* < 0.001; Fig. 6). The odds ratios for the canines compared to all other tooth types ranged from 8.6 (2.3, 32.3) to 333 (76.9, 1000). The incisors had a significantly higher probability of periodontitis at 37 weeks (p < 0.001), 32.2% (22.2, 44.0) and 78 weeks, 77.3% (66.8, 84.8), when compared to the molars and premolars with odds ratios between 3.8 (2.3, 6.4) and 35.7 (17.9, 66.7). The premolars had a significantly higher probability of periodontitis, at both 37 and 78 weeks of age, than the molars. The probability of periodontitis at 37 weeks of age for the premolars was 11.1% (6.6, 1.8) compared to 4.6% (2.2, 4.4) for the molars. At 78 weeks of age the probability of periodontitis for the premolars was 35.0% (24.6, 46.8) compared to 8.9% (4.8, 15.8) for the molars. This is an odds ratio of 2.6 (1.1, 5.8) at 37 weeks of age and 5.5 (3.0, 10.3) at 78 weeks of age. The odds of periodontitis were not significantly different between age groups for the canines (*p* = 0.38) or premolars (*p* = 0.21), but were increased for incisors and molars at 78 weeks compared to 37 weeks (*p* < 0.001). The upper jaw had a significantly higher probability of periodontitis than the lower jaw (*p* < 0.001).

**Fig. 7:**
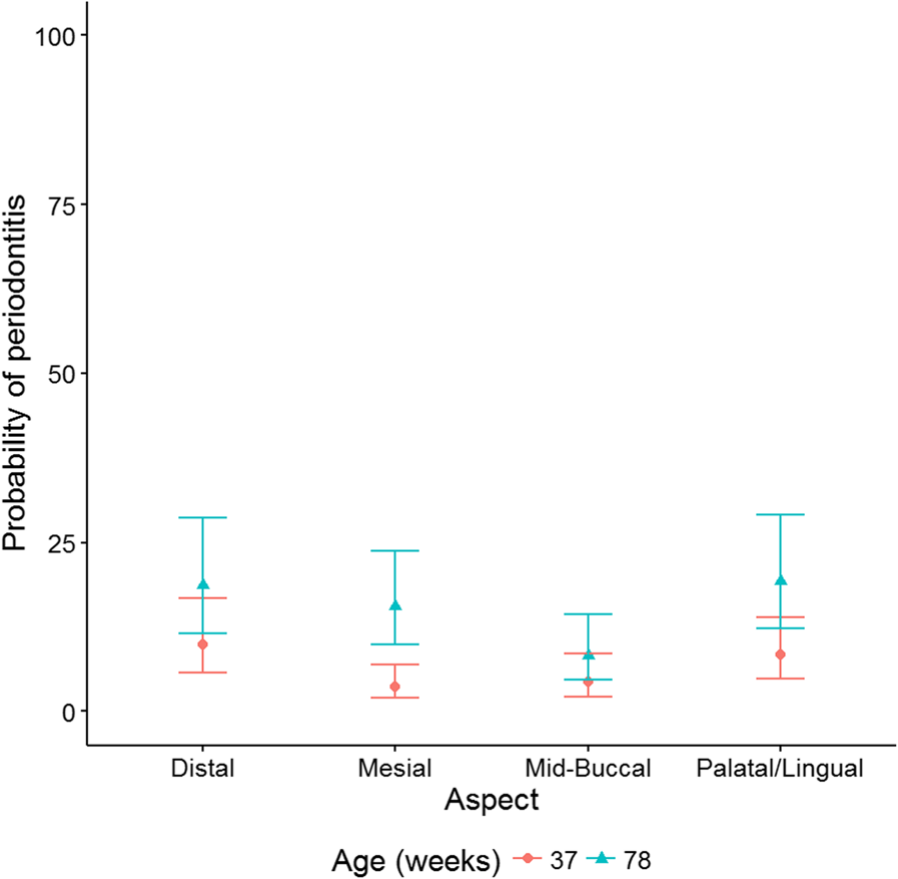
Probability of periodontitis at 37 and 78 weeks of age by aspect, with 95% confidence intervals

All aspects except the mid-buccal aspect had a significantly increased probability of periodontitis at 78 weeks compared to 37 weeks of age (*p* < 0.001; Fig. 7). The probability of periodontitis on the distal aspect was 9.9% (5.6, 17.0) at 37 weeks of age and increased to 18.6% (11.4, 29.0) at 78 weeks of age with an odds ratio of 2.1 (1.2, 3.5). The mesial aspect had a probability of periodontitis of 3.6% (1.9, 6.9) at 37 weeks which increased to 15.6% (9.7, 24.1) at 78 weeks of age with an odds ratio of 4.9 (2.6, 9.3). The palatal/lingual aspect had a probability of periodontitis of 8.3% (4.8, 14.2) at 37 weeks of age which increased to 19.3% (12.1, 29.5) at 78 weeks of age with an odds ratio of 2.6 (1.6, 4.3). With respect to the mid-buccal aspect the probability of periodontitis increased from 4.3% (2.1, 8.7) to 8.3% (4.6, 14.6), and although the odds ratio was 2.0 (1.0, 4.2), this was not significant (*p* = 0.103).

Comparison of the average mouth gingivitis score at 37 weeks of age to that at 78 weeks of age estimated that the average gingivitis score at 78 weeks of age was significantly greater than at 37 weeks of age (*p* < 0.0001). The average gingivitis score at 37 weeks was 1.48 (1.39, 1.57) and increased to 1.66 (1.57, 1.75) at 78 weeks of age, which is a difference of 0.18 (0.09, 0.28).

## Discussion

This study highlights the high prevalence of periodontal disease in Yorkshire terriers; 98% of the dogs had at least one tooth or aspect with early periodontitis at 37 weeks of age. This is higher than many of the published studies which have reported prevalence rates of periodontitis ranging from 44 to 64% in mixed age and breed populations [1–4], rising to 84% in dogs aged 3 years or older [3] to over 80% when dogs reach the age of 6 years or older [4, 5], and reaching 100% in poodles over the age of 4 years [5]. Direct comparison between studies is problematic given the differences in the criteria used to define the extent of periodontal disease, the populations included (large/small breeds, client-owned, laboratory housed etc.), the extent to which diet is considered and differences in the geographical location. A direct comparison was, however, possible between this study of Yorkshire terriers and a previous study of miniature schnauzers [12]. The dogs were housed at the same nutrition research facility, and the same criteria to define the extent of periodontal disease were used for both studies, although probing depths differed. This was to account for differences in the size of the teeth in these two breeds of dog. For the smaller Yorkshire terriers early periodontitis was defined as probing depths ≥2 mm for all teeth except the canines (≥2.5 mm), whereas probing depths of 2 mm (> 3 mm for canines) were used for the study of miniature schnauzers. In miniature schnauzers aged 1.3 to 6.9 years, 98% of dogs developed periodontitis within 60 weeks of stopping tooth brushing [12]. This affected 29% of the total number of teeth assessed. To the best of our knowledge, there has only been one other study that has demonstrated the high prevalence of periodontal disease in Yorkshire terriers compared to other breeds. Analysis of records from primary-care practices showed that Yorkshire terriers were the most affected breed with a prevalence of 25.2% compared to less than 12.8% for the other breeds investigated [11]. The reasons for the higher incidence of periodontitis in Yorkshire terriers in our study is likely due to dogs being assessed under general anaesthesia, enabling every tooth to be assessed for the very earliest stages of periodontitis around the whole gingival margin. Overall, our findings concur with other studies where a higher frequency, earlier onset and increased severity of periodontal disease has been reported in small breed dogs compared to large breeds [1, 2, 4, 10, 13]. These studies highlight that different breeds of dog have varying susceptibilities to periodontitis with smaller breeds being particularly at risk.

The average percentage of teeth with periodontitis in Yorkshire terriers increased from 25.5% at 37 weeks of age to 48.3% at 78 weeks of age, which is 2.74 times that at 37 weeks of age. Although the frequency of periodontitis teeth increased, the severity of the disease remained similar with all except one tooth classified as PD2. The average gingivitis score also increased, with an average score at 37 weeks of 1.48 compared to 1.66 at 78 weeks of age. A close relationship between age and the degree of periodontal disease in mongrel dogs, aged between 8 months and 12 years, has been shown previously [14]. In addition, a linear relationship between the age of dogs and the degree of alveolar bone loss has been demonstrated in a study of 40 beagles aged 1–8 years [15]. There have been numerous other studies that have demonstrated a correlation between age, prevalence and severity of periodontal disease [1–5, 10, 12, 16]. This study of Yorkshire terriers therefore supports previous findings in that the frequency of periodontal disease increases with age. However, measuring to 78 weeks of age is not sufficient to understand how severity progresses over a longer time period.

This study showed that the proportion of periodontitis teeth significantly differed between litters of Yorkshire terriers. Other studies have also shown that the frequency and severity of periodontal breakdown varies considerably among dogs [5, 13, 15, 17]*.* Hereditary and breed-specific forms of severe periodontal breakdown have also been reported [18], but to our knowledge there are no published studies describing the effect of litter on the prevalence of periodontitis. This finding is supported by clinical and scientific data which have indicated that there is a significant genetic influence on periodontal disease [19, 20]. It is therefore plausible to speculate that genetics will be one of the factors that partly justifies the differences in susceptibility and resistance to disease amongst breeds of dogs and individuals. However, knowledge of periodontal disease genetics is still very limited due to its polygenic nature and environmental and behavioural interactions.

The present study provides evidence of which teeth have a higher risk of developing periodontal disease in Yorkshire terriers. The canines, which developed periodontitis on all aspects of the tooth, had a higher probability of periodontitis than all other tooth types with a mean of 94.8% at 37 weeks of age. Incisors were the next most affected teeth with a probability of periodontitis of 40.2% at 37 weeks of age, and these teeth were more likely to develop periodontitis on the palatal/lingual aspect. The odds of periodontitis were not significantly different for the canines and premolars between 37 and 78 weeks but were increased for the incisors and molars. Studies of beagles, poodles and mixtures of breeds support our findings in that the canine teeth were more prone to periodontitis than any of the other types of teeth [3, 5, 10]. In miniature schnauzers however, the incisors were the most likely teeth to be affected by periodontitis, and the frequency of periodontitis on the canines was low (2% of teeth that developed periodontitis) [12]. Although incisors have not commonly been shown to be affected by periodontal disease in the published literature, several studies have reported that these teeth are one of the most likely to be mobile or lost, and in most cases it has been assumed that this is as a result of periodontitis [2, 5, 10, 16]. Other studies have reported that the most common sites of periodontal lesions are the premolars and molars [4, 16]. Although the premolars and molars of the Yorkshire terriers in this study were also affected by periodontitis, it was at a lower level than the other tooth types; 12.9 and 4.4% for premolars and molars, respectively, at 37 weeks of age, increasing to 35 and 8.9% at 78 weeks of age. The upper jaw had a significantly higher odds of periodontitis compared to the lower jaw and had a significantly quicker time to periodontitis which concurs with other studies [4, 13]. Taken together, discrepencies between the teeth affected in the various studies suggest that there are breed differences that determine which teeth are most likely to develop periodontitis.

This study had a number of limitations that could have impacted the estimates of prevalance and rates of progression of periodontal disease. Firstly, some but not all dogs received daily tooth brushing but, the Yorkshire terriers in this study were difficult to effectively tooth brush for behavioural reasons and, statistical analysis of our data showed that tooth brushing had no significant effect on the time to periodontitis. Secondly, dogs were fed a variety of diets but exploration of the impact of this in the statistical modelling showed no significant effect of diet. Thirdly, due to the constraints imposed by AWERB, teeth and dogs were removed from the study resulting in censoring of the data. This bias was accounted for in the statistical analysis by using a method that incorporates the information known about those teeth and the fact that they were still healthy at the point they left the study. These limitations, therefore, are deemed to have little affect on the estimates of prevalence and rates of progression of periodontitis reported in this study. However, other factors to consider when interpreting these data are that dogs had a full mouth professional dental cleaning (scaling and polishing) at different ages prior to their assessment at 78 weeks of age due to AWERB requirements. This is likely to have impacted the comparison between the prevalence of periodontitis at 37 compared to 78 week of age. In addition, the time to periodontitis may not be accurate due to the intervals between assessements, for example, the time to periodontitis could have been between 37 and 45 weeks of age rather than at 45 weeks of age.

Despite the limitations, this study demonstrates a high prevalence of periodontitis in Yorkshire terriers with all dogs on the study having early signs of periodontitis in one or more teeth at less than 1 year of age. It highlights that the canines and incisors are most likely to develop periodontitis first, followed by the premolars and molars. The frequency of periodontitis increased by more than two-fold over a 10-month period, and therefore it is likely that, in the absence of an effective oral care regimen, Yorkshire terriers will continue to acquire periodontitis teeth, and the severity of the disease is likely to worsen. It may be hypothesised that in Yorkshire terriers, genetic predisposition might be one of the factors that could explain the high susceptibility to periodontal disease amongst this breed.

## Conclusions

The knowledge generated from prevalence studies in different dog breeds can be used by veterinarians to improve the effectiveness of treatments by being especially vigilant with breeds, age groups and types of teeth that are at the highest risk of developing periodontitis. Veterinarians have a key role to play in educating dog owners, particularly those with susceptible breeds such as Yorkshire terriers, about the importance of effective homecare to prevent periodontal disease. Although tooth brushing remains one of the most effective methods of preventative homecare, it is not always realistic, as was found in this study. Therefore, alternative ways to retard or prevent plaque accumulation that are practical for both dogs and their owners are required.

## Methods

### Study cohort

A total of 50 Yorkshire terriers (12 litters) were enrolled onto the study. Dogs had their baseline assessment at 37 weeks of age. Yorkshire terriers were selected for the study due to the fact that small breeds of dog are more prone to periodontitis than larger breeds. Also, similar studies had already been undertaken in medium and large breed of dogs [12, 21]. This study therefore utilised a population of Yorkshire terriers that were being established at the WALTHAM Centre for Pet Nutrition enabling the study to be performed in a controlled environment where dogs could be followed longitudinally. The dogs were housed in environmentally enriched kennels and were provided with a comprehensive dog-dog and dog-human socialisation programme adjusted to their individual needs. There were 25 females and 25 males (Additional file 1: Table S1). All the males were neutered at 26 weeks of age (± 4 weeks). All the females were entire at the start of the study, but nine were spayed prior to their 78 week assessment. Their body weight at 37 weeks of age ranged from 1.63 to 8.14 kg (average 5.03 kg).

A total of 28 dogs were maintained on a commercial dry diet^1^ from weaning to 1 year of age. However, two of these dogs required the addition of diets to promote maintenance of body weight and body condition score. One dog had a commercial wet diet^2^ added to the dry diet from 41 weeks until 1 year of age. The other dog was rotated between these two diets, in combination with a veterinary diet,^3^ from 28 weeks of age until 1 year of age. A further six dogs were maintained on the commercial wet diet from 14 weeks of age. The remaining 16 dogs were fed a simultaneous offering of the dry and commercial wet diet from 14 weeks of age. After 1 year of age, dogs were fed a rotation of commercial dry and wet products formulated for small dogs.

### Study design

Dogs were recruited to the study in succession over a period of 2 years and 3 months (April 2013 to July 2015). The first 39 dogs (from 9 litters) recruited to the study were randomised across two groups by weight, gender and diet at 10 weeks of age. One group had the labial and buccal surfaces of their teeth brushed daily from 37 weeks of age, once all dogs had their permanent dentition, and the other group received no oral care regimen (Additional file 1: Table S1). Since tooth brushing was not readily accepted by all dogs, daily records were maintained confirming which areas of the mouth had been successfully brushed. Ten areas of the mouth were scored daily on a five-point scale where 1 represented an ability to ‘brush the region for at least 10 seconds’ and 5 was ‘could not brush the teeth’. Due to the general unacceptance of tooth brushing by Yorkshire terriers, the latter 11 dogs recruited to the study were not tooth brushed.

Each dog was assessed for oral health indices every 8 weeks (± 1 week), from 37 weeks up to a maximum of 61 weeks of age (see section on oral health assessment procedures). Of these, 36 were also re-assessed at 78 weeks of age.

All dogs received a pre-study veterinary examination to ensure suitability for trial which included a physical examination and an assessment of the dogs’ veterinary history. Dogs also had an examination prior to each general anaesthesia. Routine veterinary care was permitted throughout the study as required, which on occasions included administration of antibiotics and anti-inflammatory drugs. Records of these and other veterinary treatments were maintained for each dog.

### Oral health assessment procedures

Oral health assessments were performed under general anaesthesia. At the beginning of the study, the premedication used was the same as in Marshall et al. [12]. Mid-trial, the anaesthetic protocol was reviewed and is described here. EMLA cream (2.5% lidocaine and 2.5% prilocaine) (2 g) was applied on the skin over the area corresponding to the insertion of the cephalic intravenous catheter and left for 45 min. Premedication with acepromazine (0.04 mg/kg) and buprenorphine (0.04 mg/kg) was given intramuscularly, or subcutaneously at the same dose. Anaesthesia was induced with propofol (4 mg/kg) via a cephalic intravenous catheter. Following endotracheal intubation, anaesthesia was maintained with isoflurane in oxygen. All dogs received maintenance intravenous fluids (0.85% sodium chloride) at 2 ml/kg/hr. during the general anaesthesia. Cardiorespiratory parameters, mucus membrane colour, capillary refill time, temperature, eye position and palpebral/corneal reflexes, saturation of haemoglobin with oxygen (S_p_O_2_), capnography and blood pressure were measured and monitored throughout the general anaesthesia.

Four people performed the dental scoring assessments, all of whom had been trained for consistency by a Diplomate of the EVDC (Lisa Milella). Each dog was assessed by the same scorer throughout the study (with a number of unavoidable exceptions for illness). Scorers were not permitted to review previous results.

All teeth were scored individually at each assessment. Each measurement was taken at the gingival margin using a periodontal probe (14 W Williams probe). A gingivitis score between 0 and 4 was recorded for the mesial, mid-buccal, distal and palatal/lingual aspect of each tooth using a modified combination of the gingival index (GI) and sulcus bleeding index (SBI) (Table 2) [22]. The stage of periodontitis was determined by measuring and recording probing depths (rounded to the nearest 0.5 mm), gingival recession and furcation exposure, on the four aspects of each tooth outlined above, according to the criteria in Table 3. Probing depth was measured from the gingival margin to the bottom of the gingival sulcus or periodontal pocket. Gingival recession was measured from the cementoenamel junction (CEJ) to the gingival margin using the graduations of a periodontal probe. Total attachment loss was calculated as the sum of the gingival recession and the periodontal probing depth in accordance with established protocols [6, 23]. No evidence of gingival enlargement was observed. The nomenclature defined by the American Veterinary Dental College (AVDC) was used to describe the stages of periodontal disease [24]: Clinically normal with no inflammation or periodontitis (PD0), Gingivitis only with no attachment loss (PD1), early periodontitis with less than 25% attachment loss (PD2), moderate periodontitis with 25–50% attachment loss (PD3) and advanced periodontitis with greater than 50% attachment loss (PD4). As the AVDC definitions are merely descriptive, specific scoring criteria were defined for Yorkshire terriers with Diplomates of the EVDC as defined in Table 3. Dental radiographs were also taken of some of the teeth, and were inspected by a Diplomate of the EVDC to confirm the measures used in this study were indicative of early bone loss.

**Table 2:** Gingivitis (G) scoring criteria

Score	Criteria
G0	Healthy gingiva (pink or pigmented, no inflammation and no bleeding on probing)
G1	Very mild gingivitis (red, swollen but no bleeding on probing)
G2	Mild gingivitis (red, swollen and delayed bleeding on probing)
G3	Moderate gingivitis (red, swollen and immediate bleeding on probing)
G4	Severe gingivitis (ulceration, spontaneous haemorrhage, profuse bleeding on probing)

**Table 3:** Periodontitis scoring criteria used for Yorkshire terriers

AVDC Stage^a^	Periodontal Probing Depth	Gingival Recession	Furcation Exposure
Stage 2 (PD2) Early periodontitis (< 25% attachment loss)	≥2 mm (≥2.5 mm on canine teeth)	> 0 mm	Stage 1; feel an indentation between the roots and the probe may advance 1 mm.
Stage 3 (PD3) Moderate periodontitis (25–50% attachment loss)	≥4 mm (≥5 mm on canine teeth)	≥2 mm (≥3 mm on canine teeth)	Stage 2; obvious indentation between the roots and probe advances 50%.
Stage 4 (PD4) Advanced periodontitis (> 50% attachment loss)	≥8 mm (≥10 mm on canine teeth)	≥4 mm (≥6 mm on canine teeth)	Stage 3; obvious space between the roots and probe advances 100%.

^a^https://www.avdc.org/Nomenclature/Nomen-Perio.html

To avoid teeth progressing to the later stages of periodontitis, each tooth was scaled (if calculus was present) and polished as soon as periodontitis was detected and then no longer included in the study. These teeth with periodontitis were scaled (if calculus was present) and polished at every subsequent assessment until the dog was removed from the study. If a dog developed periodontitis in 12 or more teeth, it received a full mouth professional dental cleaning (scaling and polishing) and was removed from the study. These oral care criteria were selected to prevent any teeth progressing to the later stages of periodontitis. Upon completion of the trial, dogs had their oral care regimens re-instated, had their oral health status monitored every 3 months by a member of the veterinary team, and received oral care treatment as deemed necessary.

### Statistical methods

Statistical analyses were performed in R v3.2.4 [25] statistical software using libraries *lme4* [26], *multcomp* [27] and *survival* [28].

The number of dogs required for the study was determined based on the initial primary aim of investigating the effect of tooth brushing on the percentage of teeth with periodontitis in Yorkshire terriers after 6 months. A sample size calculation determined that 25 dogs were required per group to detect a 70% reduction in the proportion of periodontitis teeth, with 80% power. Specifically, a previous study of miniature schnauzers [12] was used to estimate the proportion of teeth with periodontitis after no intervention, as 12%, and the variability between dogs. This assumes the rate and variability of periodontitis will be similar in Yorkshire terriers to miniature schnauzers and that age does not affect this rate. Sample size calculations were performed by simulation of the logit linear predictor with added over dispersion according to a binomial generalised linear model analysis with tooth brushing as a fixed effect. The power was represented by the percentage of 1000 simulations where the induced effect was significant at *p* < 0.05.

An initial aim of the study was to determine the effectiveness of tooth brushing on the oral health of Yorkshire terriers. For the 14 dogs (7 litters) that were tooth brushed, and remained on the study after 37 weeks of age (Additional file 1: Table S1), data were recoded as 1 if every region of the mouth could be brushed for at least 10 s and 0 if not. The probability of success was then determined using data from 37 to 45 weeks (where the majority of dogs were still on trial). The data were analysed by GLMM, with binomial distribution with a logit link (for binary data), using penalised iterative reweighted least squares to aid convergence. Dog nested in litter was fitted as the random effects and week fitted as a fixed effect.

The average percentage of periodontitis in the mouth (dog) for each litter, at 37 weeks of age, was initially estimated by a GLMM with binomial distribution, logit link (for proportion data) and litter as a random effect. In addition, the average percentage of periodontitis in a mouth was estimated for each litter and compared between litters with a generalised linear model, with a binomial distribution, logit link (for proportion data) and litter fitted as a fixed effect. Covariates of diet (dry, wet, mixed), sex and average gingivitis in the mouth were explored as fixed effects.

The time to periodontitis for a tooth and an aspect were analysed using Accelerated Failure Time (AFT) parametric survival regression models, with a Weibull distribution and gamma frailty [28]. This method models the time to periodontitis whilst taking into account that not all teeth or aspects had the opportunity to progress to periodontitis (i.e. censored data, due to dogs coming off trial before all teeth, or aspects on a tooth, developed periodontitis). In addition, the expected correlation for repeats within a dog was allowed for by fitting dog as a random effect. Aspect, litter, tooth type, jaw, diet (dry, wet, mixed), sex, average gingivitis score on the tooth or gingivitis score on an aspect and a subset of their interactions (aspect*tooth type, aspect*jaw, aspect*gingivitis, tooth type*jaw, tooth type*gingivitis) were fitted as fixed effects as appropriate to the model (e.g. aspect would not be investigated in the tooth model). The final tooth model included tooth type, jaw location and average gingivitis score as additive fixed effects (*p* < 0.001). The final aspect model included aspect, tooth type and their interaction, along with jaw location as fixed effects (*p* < 0.001).

The probability of periodontitis in the mouth, for a tooth and an aspect, was compared from 37 weeks to 78 weeks of age using a GLMM with a binomial distribution and logit link. Tooth and aspect models used penalised iterative reweighted least squares to aid convergence. Only the data where both 37 and 78 weeks were able to be measured within a litter were included. Other missing data were assumed to be missing at random. Random effects were fitted as dog nested in litter for the mouth model and tooth nested in dog nested in litter for both the tooth and aspect models. Age, tooth type, aspect, jaw, diet (dry, wet, mixed), sex, average gingivitis in the mouth or on the tooth or gingivitis for an aspect, and a subset of their interactions (age*aspect, age*tooth type, age*gingivitis, aspect*tooth type, aspect*jaw, aspect*gingivitis, tooth type*jaw, tooth type*gingivitis) were explored as fixed effects as appropriate to the model. The final model for periodontitis in the mouth included time (*p* < 0.001) and average gingivitis (*p* = 0.048) as fixed effects. The final tooth model included the tooth type by age interaction, jaw location and average gingivitis score as fixed effects (*p* < 0.001). The final aspect model included aspect, tooth type and their interaction, along with jaw as fixed effects (*p* < 0.001).

The average gingivitis in the mouth was compared from 37 weeks to 78 weeks and was analysed by linear mixed models (LMM), with dog fitted as a random effect and age, diet group and sex explored as fixed effects. The final model only contained time as a significant fixed effect (*p* < 0.05). This type of analysis makes the assumption that the gingivitis scale is linear.

For each of the models, fixed effects were retained if significant at the 5% level, by likelihood ratio tests. Comparisons between levels within fixed effects and between interaction levels were performed by simultaneous inference to maintain family-wise error rate of 5% [27]. Average proportions, estimated time to periodontitis and average gingivitis score for the significant fixed effects and odds ratios, fold changes and differences for comparisons are reported with 95% family-wise confidence intervals.

## Additional file

Additional file 1: **Table S1.** Table detailing the 50 Yorkshire terriers recruited to the study indicating their litter, sex (M-male and F-female), tooth brushing group (NT-no tooth brushing and T-tooth brushed), diet, body weight at 37 weeks of age, number of teeth on trial and number of newly identified teeth with periodontitis at each assessment week. (DOCX 26 kb)

## Abbreviations

AFT: Accelerated Failure Time
AVDC: American Veterinary Dental College
AWERB: Animal Welfare and Ethical Review Body
CEJ: Cementoenamel junction
EVDC: European Veterinary Dental College
G0: Gingivitis level 0 (healthy gingiva)
G1: Gingivitis level 1 (very mild gingivitis)
G2: Gingivitis level 2 (mild gingivitis)
G3: Gingivitis level 3 (moderate gingivitis)
G4: Gingivitis level 4 (severe gingivitis)
GI: Gingival index
GLMM: Generalised linear mixed effects models
LMM: Linear mixed models
PD0: Periodontal disease stage 0 (clinically normal)
PD1: Periodontitis stage 1 (Gingivitis only)
PD2: Periodontitis stage 2 (early periodontitis, < 25% attachment loss)
PD3: Periodontitis stage 3 (moderate periodontitis, 25–50% attachment loss)
PD4: Periodontitis stage 4 (advanced periodontitis, > 50% attachment loss)
SBI: Sulcus bleeding index.

## Footnotes

1 Royal Canin Yorkshire Terrier Puppy 29™

2 Pedigree® puppy

3 Royal Canin recovery®
