# Supplementary material for: A longitudinal assessment of periodontal disease in Yorkshire terriers
Source: BMC Vet Res. 2019 Jun 21;15:207. doi: 10.1186/s12917-019-1923-8 (PMC6588847; doi:10.1186/s12917-019-1923-8)
Supplement: Supplementary file 1 — Table S1. Table detailing the 50 Yorkshire terriers recruited to the study indicating their litter, sex (M-male and F-female), tooth brushing group (NT-no tooth brushing and T-tooth brushed), diet, body weight at 37 weeks of age, number of teeth on trial and number of newly identified teeth with periodontitis at each assessment week. (DOCX 26 kb) [file 12917_2019_1923_MOESM1_ESM.docx]

# **Supplementary Table 1.** Table detailing the 50 Yorkshire terriers recruited to the study indicating their litter, sex (M-male and F-female), tooth brushing group (NT-no tooth brushing and T-tooth brushed), diet, body weight at 37 weeks of age, number of teeth on trial and number of newly identified teeth with periodontitis at each assessment week.

| **Dog** | **Litter** | **Sex** | **Tooth brushing Group** | **Diet** | **Body weight (Kg)** | **Number of on trial teeth at each assessment week** | | | | | **Number of periodontitis teeth at each assessment week** | | | | |
| --- | --- | --- | --- | --- | --- | --- | --- | --- | --- | --- | --- | --- | --- | --- | --- |
|  |  |  |  |  |  | **37** | **45** | **53** | **61** | **78** | **37** | **45** | **53** | **61** | **78** |
| A1 | A | F | NT | Dry | 4.60 | 38 | 30 |  |  | 38 | 8 | 11 |  |  | 21 |
| A2 | A | F | T | Mixed | 5.85 | 40 | 32 |  |  | 40 | 8 | 7 |  |  | 14 |
| A3 | A | M | NT | Mixed | 5.50 | 38 | 33 | 30 |  | 38 | 4 | 4 | 23 |  | 20 |
| A4 | A | M | T | Dry | 7.02 | 41 |  |  |  | 41 | 16 |  |  |  | 16 |
| A5 | A | F | T | Wet | 5.49 | 42 |  |  |  | 42 | 14 |  |  |  | 17 |
| A6 | A | M | NT | Wet | 6.30 | 40 |  |  |  | 40 | 14 |  |  |  | 25 |
| C1 | C | F | T | Dry | 6.84 | 34 |  |  |  | 35 | 12 |  |  |  | 15 |
| C2 | C | M | NT | Dry | 6.32 | 37 | 35 |  |  | 37 | 2 | 14 |  |  | 20 |
| C3 | C | M | T | Wet | 7.60 | 39 | 38 |  |  | 39 | 0 | 13 |  |  | 15 |
| C4 | C | M | NT | Mixed | 6.42 | 41 |  |  |  | 41 | 12 |  |  |  | 27 |
| C5 | C | F | NT | Wet | 7.72 | 35 | 28 |  |  | 35 | 7 | 5 |  |  | 21 |
| C6 | C | M | T | Dry | 6.44 | 36 | 26 |  |  | 36 | 10 | 4 |  |  | 20 |
| C7 | C | M | NT | Wet | 8.14 | 38 | 33 |  |  | 38 | 5 | 7 |  |  | 20 |
| C8 | C | F | T | Mixed | 4.34 | 36 | 31 |  |  | 36 | 5 | 8 |  |  | 17 |
| E1 | E | M | None | Mixed | 5.46 | 40 |  |  |  |  | 19 |  |  |  |  |
| E2 | E | M | None | Mixed | 5.36 | 40 |  |  |  |  | 14 |  |  |  |  |
| E3 | E | M | None | Mixed | 7.08 | 42 |  |  |  |  | 18 |  |  |  |  |
| F1 | F | M | None | Mixed | 5.80 | 37 |  |  |  |  | 15 |  |  |  |  |
| F2 | F | F | None | Mixed | 5.20 | 41 |  |  |  |  | 17 |  |  |  |  |
| F3 | F | M | None | Mixed | 7.00 | 40 |  |  |  |  | 22 |  |  |  |  |
| F4 | F | M | None | Mixed | 7.52 | 41 | 30 |  |  |  | 11 | 14 |  |  |  |
| F5 | F | M | None | Mixed | 7.68 | 36 |  |  |  |  | 16 |  |  |  |  |
| I1 | I | F | None | Mixed | 2.04 | 35 |  |  |  |  | 20 |  |  |  |  |
| I2 | I | F | None | Mixed | 1.96 | 32 |  |  |  |  | 13 |  |  |  |  |
| I3 | I | F | None | Mixed | 2.26 | 36 |  |  |  |  | 24 |  |  |  |  |
| Q1 | Q | F | NT | Dry | 3.40 | 37 | 29 | 27 |  | 38 | 9 | 2 | 12 |  | 24 |
| Q2 | Q | F | NT | Dry | 1.63 | 40 | 33 |  |  | 40 | 7 | 16 |  |  | 28 |
| Q3 | Q | F | T | Dry | 2.05 | 38 | 31 | 18 |  | 41 | 8 | 13 | 7 |  | 23 |
| Q4 | Q | F | T | Dry | 1.49 |  |  |  |  | 40 |  |  |  |  | 29 |
| R1 | R | F | NT | Dry | 2.14 | 36 | 23 |  |  | 36 | 13 | 4 |  |  | 8 |
| R2 | R | F | T | Dry | 1.97 | 37 | 27 | 27 |  | 37 | 12 | 0 | 10 |  | 12 |
| R3 | R | M | T | Dry | 2.58 | 40 | 34 | 30 |  | 40 | 6 | 4 | 4 |  | 10 |
| R4 | R | F | NT | Dry | 2.25 | 40 | 28 | 25 | 24 | 40 | 12 | 3 | 1 | 7 | 15 |
| S1 | S | M | T | Dry | 4.62 | 42 | 34 |  |  | 42 | 8 | 10 |  |  | 29 |
| S2 | S | F | NT | Dry | 3.28 | 39 | 30 |  |  |  | 9 | 8 |  |  |  |
| S3 | S | F | T | Dry | 2.26 | 39 | 29 |  |  | 39 | 10 | 9 |  |  | 16 |
| S4 | S | M | T | Dry | 2.26 |  | 33 |  |  |  |  | 15 |  |  |  |
| S5 | S | F | NT | Dry | 2.26 | 40 | 29 |  |  | 40 | 11 | 8 |  |  | 19 |
| U1 | U | M | NT | Dry | 8.04 | 42 | 33 |  |  | 42 | 9 | 7 |  |  | 24 |
| U2 | U | M | T | Dry | 6.19 | 39 | 32 |  |  | 39 | 7 | 6 |  |  | 22 |
| U3 | U | M | T | Dry | 4.31 | 35 |  |  |  |  | 19 |  |  |  |  |
| U4 | U | M | NT | Dry | 7.31 | 40 | 36 |  |  | 41 | 5 | 9 |  |  | 23 |
| V1 | V | M | T | Dry | 5.04 | 36 |  |  |  | 35 | 21 |  |  |  | 21 |
| V2 | V | M | T | Dry | 5.84 | 40 |  |  |  | 40 | 19 |  |  |  | 19 |
| V3 | V | M | NT | Dry | 4.35 | 39 |  |  |  | 40 | 15 |  |  |  | 18 |
| V4 | V | F | NT | Dry | 5.09 | 40 |  |  |  | 40 | 14 |  |  |  | 29 |
| V5 | V | F | T | Dry | 5.17 | 36 |  |  |  |  | 13 |  |  |  |  |
| V6 | V | F | NT | Dry | 5.65 | 35 |  |  |  | 34 | 16 |  |  |  | 22 |
| Y1 | Y | F | NT | Wet | 6.18 | 38 | 32 |  |  | 40 | 8 | 21 |  |  | 14 |
| Y2 | Y | F | T | Dry | 6.81 | 33 | 25 |  |  | 33 | 8 | 7 |  |  | 16 |
| Z1 | Z | F | T | Mixed | 4.68 | 38 | 32 | 27 |  | 38 | 6 | 5 | 6 |  | 13 |
| **Total** | | | | | | **1874** | **866** | **184** | **24** | **1351** | **571** | **234** | **63** | **7** | **673** |
